# Association between the cholesterol-high-density lipoprotein-glucose index and new-onset stroke in middle-aged and older adults with depressive symptoms: a longitudinal cohort analysis

**DOI:** 10.1186/s12944-026-03001-5

**Published:** 2026-07-18

**Authors:** Yishu Wen, Wei Zhang, Lanxiang Wu, Jiaqi Zhu, Yingxue Yu, Xinping Yu, Sheng Tian, Qi Zuo, Runyu Zhou, Mingxu Liu, Wei Wu, Heqing Zheng

**Affiliations:** 1 Department of Neurology, The Second Affiliated Hospital, Jiangxi Medical College, Nanchang University, 1# Minde Road, Nanchang, Jiangxi Province 330006 P. R. China; 2 Department of Rehabilitation Medicine, The Second Affiliated Hospital, Jiangxi Medical College, Nanchang University, 1# Minde Road, Nanchang, Jiangxi Province 330006 P. R. China

**Keywords:** CHG index, Depression, Stroke, CHARLS, China

## Abstract

**Background:**

Depression is a global disease that significantly increases the risk of stroke. The cholesterol, high-density lipoprotein, and glucose index (CHG index) as an emerging composite metabolic marker has shown value in cardiovascular diseases and diabetes, but its predictive role in new-onset stroke among depressed individuals remains unclear.

**Methods:**

This study is based on the data from the Chinese Health and Retirement Longitudinal Study (CHARLS) from 2011 to 2018. A total of 5,138 participants aged 45 and above, who had depressive symptoms (CES-D score ≥ 10) at the baseline and had no history of stroke, were included. Sequential Cox proportional hazards regression models were used as the main analysis to evaluate the association between the CHG index and new-onset stroke, and multivariable logistic regression was used as a secondary analysis to verify the consistency of the direction. Multiple imputation (20 datasets) was used to handle missing values of covariates. Restricted cubic splines and two-stage Cox regression models were used to explore potential nonlinear relationships and threshold effects.

**Results:**

During a total follow-up period of 35,554 person-years (with a median follow-up of 7 years), a total of 421 new-onset stroke events occurred. In the fully adjusted Cox model, for every 1-unit increase in the CHG index, the risk of new-onset stroke increased by 48% (HR = 1.48, 95% CI: 1.15–1.90, *P* = 0.003). However, the restricted cubic spline analysis revealed a significant non-linear relationship (D1 test *P* = 0.005), with the inflection point occurring at a CHG index of 5.59. When the CHG index was below 5.59, for every 1-unit increase, the risk of stroke increased by 117% (HR = 2.17, 95% CI: 1.37–3.44, *P* = 0.001); above this inflection point, no statistically significant association was observed (HR = 1.00, 95% CI: 0.62–1.62, *P* = 0.996). Multivariable logistic regression yielded a consistent significant association (OR = 1.53, 95% CI: 1.16–2.03, *P* = 0.003). Subgroup analysis and multiple sensitivity analyses (quartile analysis, exclusion of early stroke events, median binary classification analysis) all supported the robustness of the main findings.

**Conclusion:**

Among middle‑aged and older Chinese adults with depressive symptoms, the CHG index shows a significant non-linear positive association with the risk of new-onset stroke, and the risk increases sharply when the index is below 5.59. The CHG index may serve as a potential indicator for the early risk of stroke in this high-risk population.

**Supplementary Information:**

The online version contains supplementary material available at https://doi.org/10.1186/s12944-026-03001-5.

## Introduction

Depression has become a major public health issue worldwide. According to the Global Burden of Disease study (GBD) in 2017, 260 million people were suffering from depression [1, 2]. The World Health Organization predicts that by 2030, major depressive disorder will become the leading cause of disease burden worldwide [3]. The number of people suffering from depression in China accounts for 21.3% of the global total. Depression has become a significant factor in China’s disease burden [1]. Previous studies have shown that depression is a significant risk factor for cardiovascular and cerebrovascular diseases, significantly increasing the risk of onset and poor prognosis [4, 5].

Stroke is the most severe cerebrovascular illness and the primary cause of long-term impairment in adults. It is also the second greatest cause of mortality globally [6, 7]. In China, stroke has become the leading cause of death among chronic diseases and a major public health burden [8, 9]. For clinical therapy and prognosis management, early anticipated biomarkers are thus very important. The risk of stroke is now linked to homocysteine, C-reactive protein (CRP), total cholesterol (TC), and high-density lipoprotein cholesterol (HDL‑C) [10–13]. However, there are multiple pathological mechanisms interacting with each other in stroke, and a single biological indicator may not be able to provide more precise guidance for clinical decisions. Therefore, the search for composite biomarkers is of vital importance for preventing stroke and alleviating its associated social burden.

Studies have shown that the CHG index is significantly associated with cardiovascular diseases, type 2 diabetes, and IgA nephropathy. CHG index is an emerging composite metabolic marker that integrates total cholesterol (TC), fasting blood glucose (FBG) and high-density lipoprotein cholesterol (HDL‑C) into a single measurement indicator. The CHG index can provide a more comprehensive reflection of lipid and glucose metabolic disorders than a single indicator, and may also reflect the damage of these metabolic pathways to target organs [14–16]. However, at present, the relationship between the CHG index and the occurrence of new-onset stroke among middle-aged and elderly individuals with depressive symptoms is still unclear.

Therefore, this study utilized the Chinese Health and Retirement Longitudinal Study (CHARLS) database to assess the relationship between the CHG index and new-onset stroke in the population with depressive symptoms, including evaluating potential nonlinear relationships and inflection points, in order to provide a more comprehensive biomarker for clinical practice.

## Materials and methods

### Data source and research sample

This research data is sourced from the CHARLS database. CHARLS is a survey that tracks the population aged 45 and above in the Chinese mainland. Its baseline survey began in 2011 and regular questionnaire tracking surveys are conducted every two years. The survey ended in 2020, involving 17,708 individuals and covering 150 regions and 450 communities across the country. Moreover, CHARLS is based on stratified random sampling, with a 80.5% response rate for the baseline survey, which helps reduce selection bias and ensures that the research sample has good national representativeness in terms of demographic characteristics [17].

The inclusion and measurement of the participants with depressive symptoms were conducted by using the 10-item Center for Epidemiologic Studies Depression Scale (CESD-10) from the CHARLS survey to assess depression. This tool has been fully validated in the elderly population in China [18]. The CESD-10 consists of 10 questions, among which two are positive emotion items. The options are scored on a scale of 4 points: “Rarely or never, < 1 day”, “Some or a little of the time, 1–2 days”, “Occasionally or a moderate amount of the time, 3–4 days”, and “Most or all of the time, 5–7 days”. The positive emotion items are scored inversely. The scores of all 10 items are summed up, and the total score ranges from 0 to 30. When the total score is ≥ 10, the participant is considered to have depression [19].

**Fig. 1:**
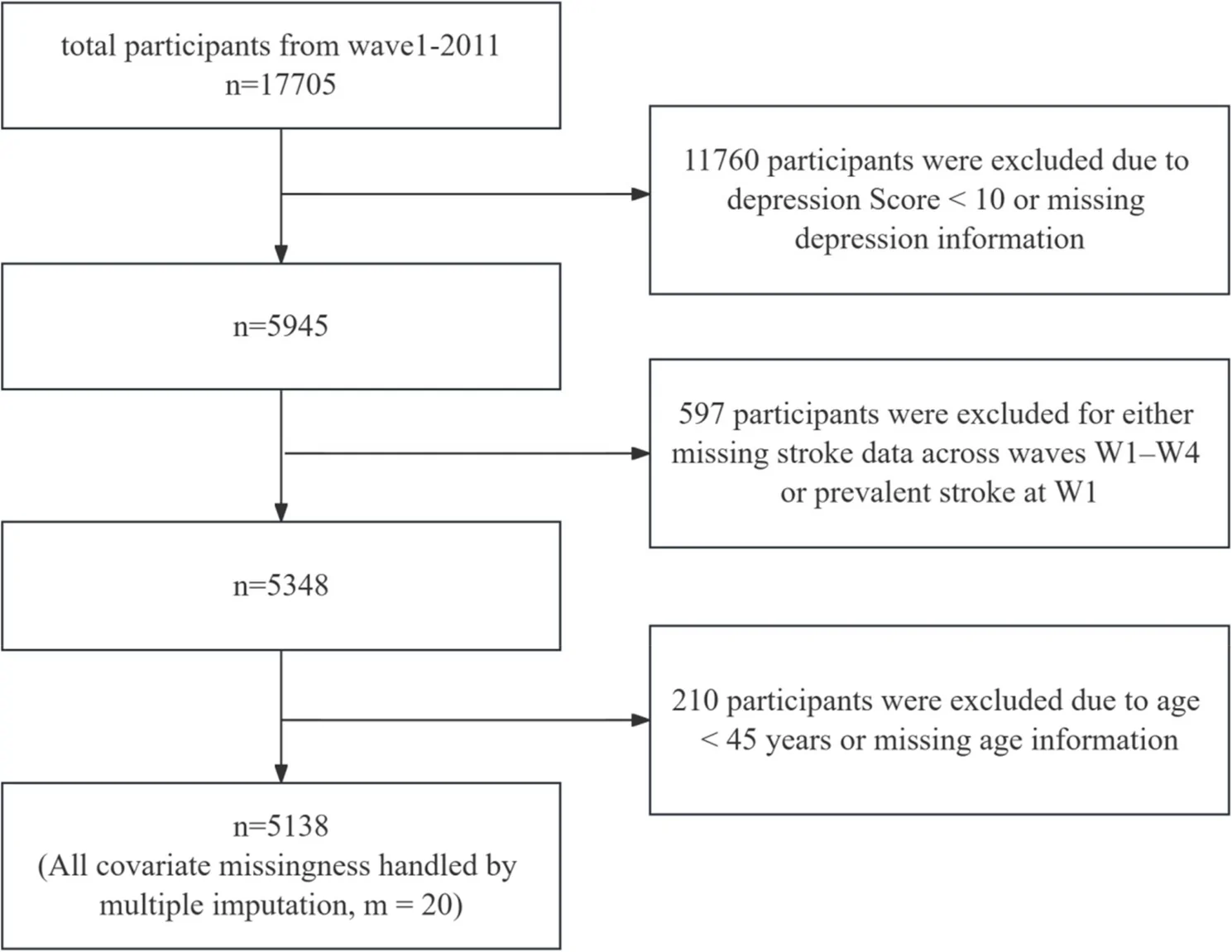
Flowchart of sample selection

We analyzed the baseline data from 2011 and the follow-up data from 2013, 2015, and 2018. Among the 17,705 baseline participants, we screened them according to the following criteria: [1] age ≥ 45 years; [2] no stroke at baseline; [3] CESD-10 score ≥ 10 and no missing values; [4] at least one follow-up available for the new-onset stroke outcome data. A total of 5,138 participants who met these criteria and were included in the multiple imputation analysis framework were identified. For the missing values in covariates (such as 34.8% missing for CHG and 14.1% missing for BMI, etc.), we used multiple imputation by chained equations (MICE) to generate 20 complete data sets. All analyses were conducted on the imputed data and the effect sizes were combined using the Rubin rule. The detailed process of excluding participants is shown in Fig. 1.

## Research variables

### Definition of Cholesterol, high-density Lipoprotein, and Glucose Index

The CHG index, as an emerging metabolic marker, effectively integrates lipid and blood sugar information, and can comprehensively assess metabolic risks more accurately than a single indicator. In this study, the data of the CHG index originated from the blood sample test results of the CHARLS database. It adopted a standardized process for blood collection, cold chain transportation, and laboratory testing. All samples were analyzed at the cooperative laboratory of Peking University (KingMed Diagnostics), and its testing method met international quality control standards. The Between-Assay Coefficient of Variation (CV) for Total Cholesterol (TC), Fasting Blood Glucose (FBG), and high-density Lipoprotein Cholesterol (HDL‑C) were all below 5%, ensuring the reliability and consistency of the data [20]. The calculation formula for CHG index is ln[ (TC (mg/dL) × FBG (mg/dL)) / (2 × HDL‑C (mg/dL)) ] [14]. Where TC, FBG, and HDL‑C denote total cholesterol, fasting blood glucose, and high-density lipoprotein cholesterol, respectively. Lipid parameters were converted from mmol/L to mg/dL by multiplying by 38.67, and glucose by 18.02.

### Definition of endpoint event

The endpoint event of this study is “new-onset stroke”, defined as the first occurrence of a stroke event that was self-reported and diagnosed by a doctor during the follow-up period, including cerebral infarction and cerebral hemorrhage. The endpoint event is confirmed through a series of questions in the CHARLS questionnaire. Firstly, “Has a doctor ever told you that you had a stroke?” is used to exclude participants who had previously suffered from a stroke. On this basis, a “new-onset stroke” event is considered when the participant gives a positive answer for the first time to “Have you been diagnosed with a stroke by a doctor since your last follow-up?” in a non-baseline follow-up. “When was the stroke diagnosed or did you know about it yourself?” or “When was your last stroke?” is used to confirm the time of the event. All participants entered the follow-up starting from the baseline survey in 2011 until they experienced a new-onset stroke, died, were lost to follow-up, or until the end of the fourth round of visits in 2018. These operations ensure the accuracy of endpoint event identification and the precision of time determination, providing a reliable basis for subsequent analysis of the association between CHG Index and new-onset stroke.

### Covariate variables

We selected covariates that might be associated with new-onset stroke in patients with depression, including sociodemographic characteristics, lifestyle behaviors, clinical measurement indicators, and depression assessment indicators. Sociodemographic characteristics included age (45 years and above), gender (female/male), place of residence (Urban community/Rural village), marital status (Married/Separated/divorced/widowed and never married), and educational level (Elementary school or below, Middle school to high school, Vocational school, college and above). Lifestyle behaviors included smoking status (yes/no) and drinking status (yes/no) [21, 22]. Clinical measurement indicators included the CHG index (mean ± standard deviation), as well as self-reported history of liver disease (yes/no) and history of digestive system diseases (yes/no) [23].Additionally, we included history of hypertension (yes/no) [24], history of diabetes (yes/no), history of psychotherapy (yes/no), and body mass index (BMI, classified as normal weight, overweight, and obese) [25], to more comprehensively control metabolic confounding factors. Depression assessment was measured by the CES-D score (score ≥ 10). Variables with missing values among all covariates were handled through multiple imputation based on the chain equation (MICE, 20 imputation data sets), with the imputation model including all the above covariates and the outcome variable (new-onset stroke).

### Statistical analysis

All statistical analyses in this study were conducted using the R software (version 4.1.3), with the two-sided significance level set at α = 0.05. The study population was divided into two groups based on whether a new-onset stroke occurred, and their baseline characteristics were described. Continuous variables that followed a normal distribution were expressed as mean ± standard deviation (Mean ± SD), and comparisons between groups were performed using the independent sample t-test; continuous variables that did not follow a normal distribution were expressed as median (interquartile range), and comparisons between groups were conducted using the Mann–Whitney U test. Categorical variables were expressed as frequency (percentage), and comparisons between groups were performed using the chi-square test or Fisher’s exact probability method. To assess the selection bias, we compared the basic characteristics of the 5138 participants who were ultimately included in the analysis with those of the 12,567 participants who were excluded based on any screening criteria (Supplementary Table S1).

Although the CHG index was only officially named and validated in 2025, its components, total cholesterol (TC), fasting blood glucose (FBG), and high-density lipoprotein cholesterol (HDL‑C), have been routinely measured for decades and were also documented in CHARLS baseline blood data in 2011. Therefore, the CHG index was calculated retrospectively, and this analysis should be regarded as an exploratory analysis. Given the longitudinal design of this study and the time-event nature of the follow-up data, we employed the Cox proportional hazards regression model as the main analysis to assess the association between the CHG index and the occurrence of new-onset strokes. The follow-up period was defined as from the baseline in 2011 to the first occurrence of stroke, death, loss to follow-up, or the end of the 2018 follow-up (corresponding to three follow-up waves of 2 years, 4 years, and 7 years respectively). To evaluate the incremental impact of covariate adjustment, three sequential Cox proportional hazards models were constructed. Model 1 was adjusted for age and sex only. Model 2 additionally adjusted for marital status, education level, smoking status, and drinking status. Model 3 (fully adjusted) further included liver disease, digestive system disease, CES‑D score, history of hypertension, history of diabetes, history of psychological treatment, and BMI classification. Liver disease and digestive diseases were included because of their documented associations with systemic inflammation, metabolic syndrome, and stroke risk [23]. Results are presented as hazard ratios (HR) with 95% confidence intervals (CI). As a secondary analysis, we also constructed a logistic regression model to test the consistency of the direction of the association.

To address the missing data in the covariates (where CHG was missing by 34.8%, BMI was missing by 14.1%, and the missing rates for the other variables were all less than 1%), we employed a multiple imputation method based on the chain equation to generate 20 complete data sets. The imputation model included all the aforementioned covariates as well as the outcome variable (new-onset stroke). All regression analyses were conducted separately on each imputed data set, and the effect estimates were combined using the Rubin rules.

To further explore the possible non-linear relationship between the CHG index and new-onset stroke, a restricted cubic spline (RCS) model was used for fitting. The nodes were set at the 10th percentile, 50th percentile, and 90th percentile of the CHG index distribution. Within the framework of multiple imputation, we separately fitted linear models and non-linear RCS models for each imputed dataset, and used the D1 statistic for likelihood ratio tests to evaluate the overall significance of the non-linear components. Additionally, in the fully adjusted model, two-segment linear regression was employed to determine the potential turning points, and the two-segment model was compared with the standard linear model through likelihood ratio tests to assess the statistical significance of the threshold effect. We estimated the thresholds in 20 imputed datasets, took the median, and reported the 2.5%–97.5% percentiles. Furthermore, 200 Bootstrap resampling was conducted on a representative imputed dataset, and the 95% confidence interval of the threshold was estimated. The two‑piecewise Cox regression was performed under Model 3 (fully adjusted) to identify the threshold effect. The HR and 95% CI were calculated separately for CHG values below and above the inflection point. Although the primary inference was based on the non-linear analyses (restricted cubic splines and two-piecewise Cox regression), we also retained the conventional continuous association (hazard ratio per 1-unit increase) as a supplementary reference. This simplified summary measure facilitates comparison with previously published linear estimates of similar metabolic indices.

We conducted subgroup analyses to evaluate the consistency of the association between the CHG index and new-onset stroke in different population subgroups. The subgroups were classified based on age (< 60 years / ≥ 60 years), gender, place of residence (urban community / rural village), smoking status (yes/no), drinking status (yes/no), history of hypertension (yes/no), history of diabetes (yes/no), and BMI classification (normal weight / overweight / obese). The results of the subgroup analysis were presented in the form of a forest plot.

To verify the robustness, we conducted a sensitivity analysis. Firstly, the CHG index was divided into quartiles (Q1–Q4). The cutoff values and baseline characteristics of participants in each quartile are provided in Supplementary Table S2. A multivariable Cox regression model was used to assess the association between each quartile and the occurrence of new-onset stroke. The lowest quartile (Q1) was set as the reference group, and the linear trend was tested by inputting the median of each quartile as a continuous variable into the regression model. Secondly, after excluding participants who had experienced stroke within the first 2 years of follow-up, the main Cox regression analysis was repeated to reduce the interference of potential reverse causality on the results. Additionally, we also conducted a binary classification analysis using the pre-defined clinical cutoff value (median) to avoid a single reliance on data-driven thresholds.

## Results

### Baseline characteristics of the study population

A total of 5,138 participants with depressive symptoms (CES-D score ≥ 10 points) were included in this study. The median follow-up period was 7 years (from 2011 to 2018). During the 35,554 person-years of follow-up, 421 new-onset stroke events were recorded (incidence rate 11.8/1,000 person-years). As shown in Table 1, compared with the non-stroke group, the baseline CHG index of patients in the new-onset stroke group was significantly higher (median 5.39 [IQR: 5.11–5.64] vs. 5.26 [5.00–5.51], *P* < 0.001). In addition, patients in the new-onset stroke group were older (60.86 ± 8.74 years vs. 59.57 ± 9.48 years, *P* = 0.007), had a higher prevalence of hypertension (46.4% vs. 25.9%, *P* < 0.001), a higher prevalence of diabetes (11.7% vs. 6.2%, *P* < 0.001), a higher CES-D score (median 15.00 vs. 14.00, *P* = 0.001), and a slightly higher BMI (median 23.55 vs. 22.53, *P* < 0.001). There were no statistically significant differences in gender, place of residence, marital status, education level, smoking status, drinking status, history of digestive system diseases, and history of psychological treatment between the two groups (all *P* > 0.05). There was a statistically significant difference in the history of liver disease between the two groups (6.7% vs. 4.4%, *P* = 0.038), but the overall prevalence was lower.

**Table 1:** The baseline characteristics of participants in this study

Variable	Total(*n* = 5138)	Non-new-onset stroke (*n* = 4717)	new-onset stroke (*n* = 421)	*p*
Age, Mean ± SD	59.67 (9.43)	59.57 (9.48)	60.86 (8.74)	0.007
Gender, *n* (%)				0.498
Female	3197 (62.2)	2942 (62.4)	255 (60.6)	
Male	1941 (37.8)	1775 (37.6)	166 (39.4)	
Residence, *n* (%)				0.191
Urban community	1606 (31.3)	1462 (31.0)	144 (34.2)	
Rural village	3532 (68.7)	3255 (69.0)	277 (65.8)	
Marital status, *n* (%)				0.156
Married	4340 (84.5)	3995 (84.7)	345 (81.9)	
Separated, divorced, widowed and never married	798 (15.5)	722 (15.3)	76 (18.1)	
Education, *n* (%)				0.412
Elementary school or below	2916 (56.8)	2689 (57.0)	227 (53.9)	
Middle school to high school	2134 (41.5)	1949 (41.3)	185 (43.9)	
Vocational school, college and above	88 ( 1.7)	79 ( 1.7)	9 ( 2.1)	
Smoking, *n* (%)				0.121
No	3368 (65.6)	3107 (65.9)	261 (62.0)	
Yes	1770 (34.4)	1610 (34.1)	160 (38.0)	
Drinking, *n* (%)				0.236
No	3357 (65.4)	3094 (65.6)	263 (62.6)	
Yes	1778 (34.6)	1621 (34.4)	157 (37.4)	
Liver disease, *n* (%)				0.038
No	4862 (95.4)	4472 (95.6)	390 (93.3)	
Yes	232 ( 4.6)	204 ( 4.4)	28 ( 6.7)	
Digestive disease, *n* (%)				1.000
No	3550 (69.3)	3258 (69.3)	292 (69.4)	
Yes	1574 (30.7)	1445 (30.7)	129 (30.6)	
CES-D score (median [IQR])	14.00 [12.00, 18.00]	14.00 [11.00, 18.00]	15.00 [12.00, 19.00]	0.001
Hypertension, *n* (%)				< 0.001
No	3696 (72.4)	3471 (74.1)	225 (53.6)	
Yes	1409 (27.6)	1214 (25.9)	195 (46.4)	
Diabetes, *n* (%)				< 0.001
No	4751 (93.3)	4382 (93.8)	369 (88.3)	
Yes	339 ( 6.7)	290 ( 6.2)	49 (11.7)	
Psychological treatment, *n* (%)				0.800
No	5102 (99.7)	4684 (99.7)	418 (99.5)	
Yes	15 ( 0.3)	13 ( 0.3)	2 ( 0.5)	
BMI (median [IQR])	22.62 [20.31, 25.33]	22.53 [20.24, 25.22]	23.55 [21.08, 26.24]	< 0.001
CHG (median [IQR])	5.27 [5.01, 5.53]	5.26 [5.00, 5.51]	5.39 [5.11, 5.64]	< 0.001

Continuous variables are shown as Mean (SD) or Median [IQR]; categorical variables as n (%). *P* values from t-test / Kruskal-Wallis / chi-square test. Data from the first imputed dataset. Total person-years = 35,554; median follow-up = 7 years; new-onset stroke rate = 11.8 per 1,000 person-years

*Abbreviations*: *BMI* Body mass index, *CES‑D* Center for Epidemiologic Studies Depression Scale, *CHG* Cholesterol–high‑density lipoprotein–glucose index, *IQR* Interquartile range, *SD* Standard deviation

### The association between CHG index and new-onset stroke

**Fig. 2:**
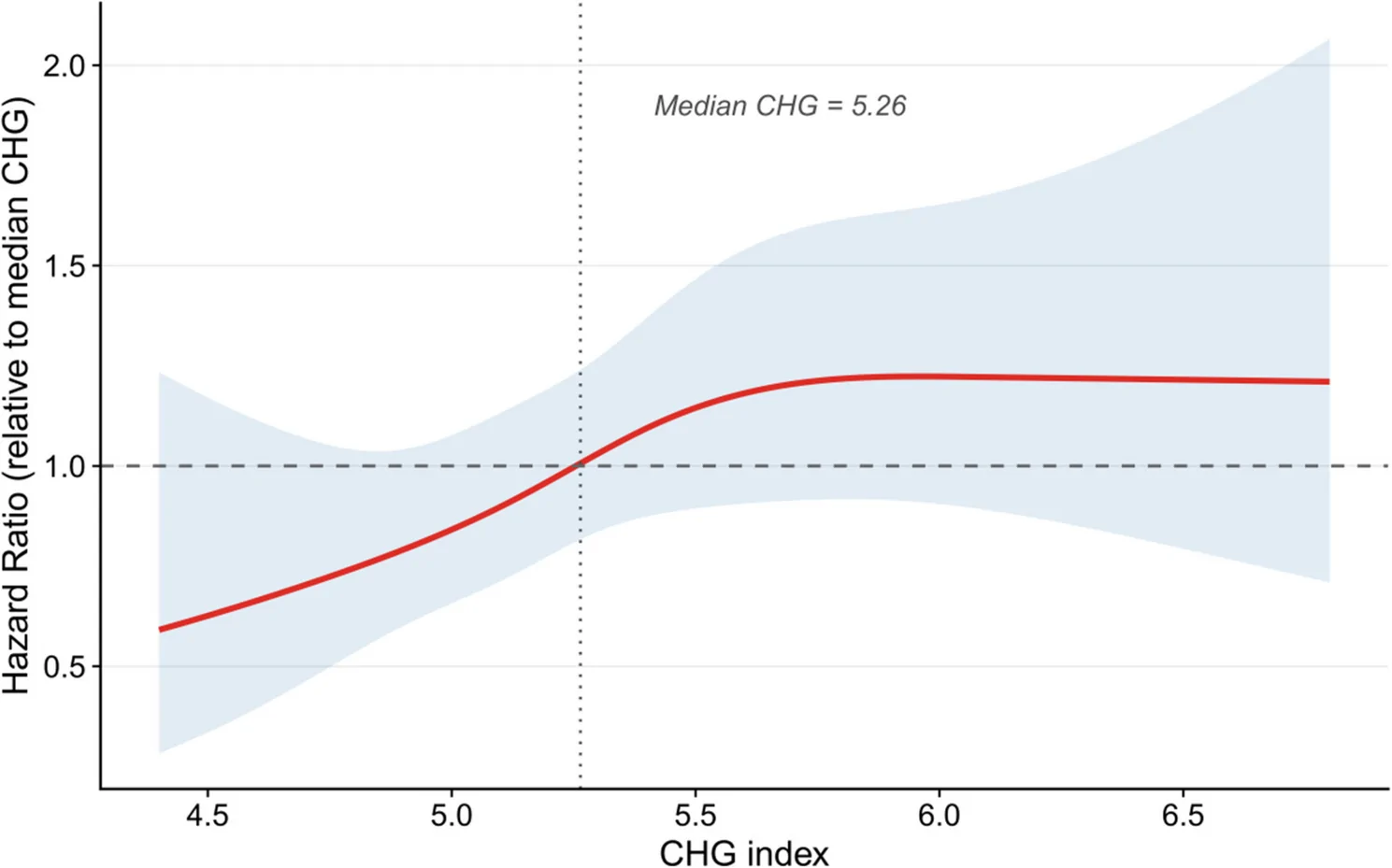
Restricted cubic spline curve for the association between CHG index and new-onset stroke in the fully adjusted Cox proportional hazards model. The solid red line represents the fitted nonlinear relationship, and the shaded area indicates the 95% confidence interval. The curve is centered at the median CHG index (5.26, dotted vertical line), which serves as the reference point (HR = 1). The dashed vertical line marks the inflection point (CHG index = 5.59) identified by two-piecewise Cox regression. The model has been adjusted for age, gender, residence, marital status, education level, smoking, drinking, liver disease, digestive disease, CES-D score, hypertension, diabetes, psychological treatment, and BMI classification

**Fig. 3:**
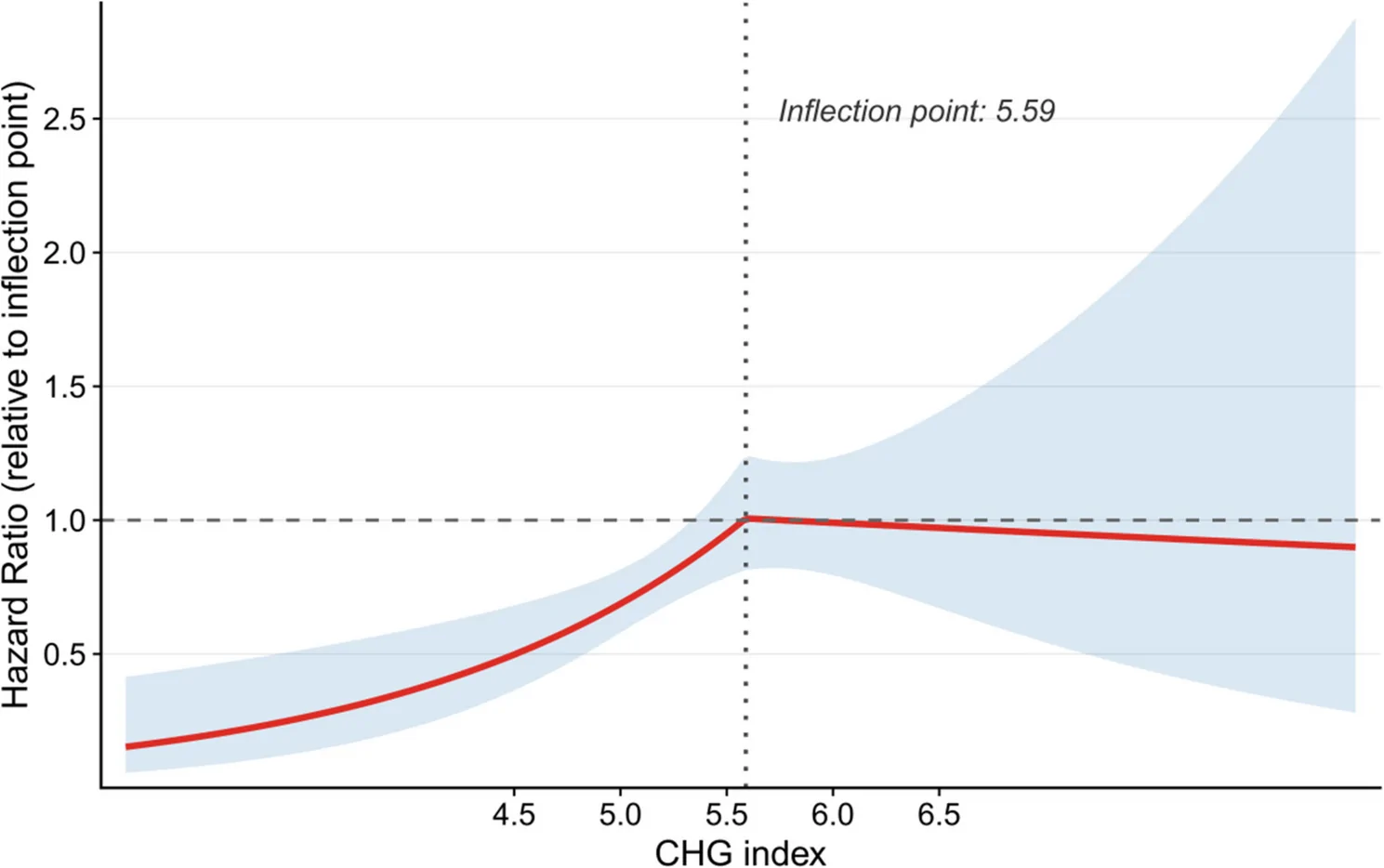
Two-piecewise Cox regression for the association between CHG index and new-onset stroke. The solid red line represents the predicted hazard ratio (HR) relative to the inflection point (CHG index = 5.59, vertical dashed line), and the shaded area indicates the 95% confidence interval. Below the inflection point, stroke risk increased sharply with increasing CHG index; above the inflection point, the association plateaued. The model was adjusted for age, gender, residence, marital status, education level, smoking, drinking, liver disease, digestive disease, CES-D score, hypertension, diabetes, psychological treatment, and BMI category. Results were pooled across 20 multiply imputed datasets

For reference purposes, we examined the conventional linear association across three sequential Cox models (Table 2). In Model 1 (adjusted for age and sex only), each 1‑unit increase in CHG was associated with an 84% higher stroke risk (HR = 1.84, 95% CI: 1.49–2.27, *P* < 0.001). Additional adjustment for socioeconomic and lifestyle factors (Model 2) did not materially change the estimate (HR = 1.84, 95% CI: 1.49–2.26, *P* < 0.001). In the fully adjusted model (Model 3), the association attenuated but remained statistically significant (HR = 1.48, 95% CI: 1.15–1.90, *P* = 0.003). Importantly, the restricted cubic spline analysis revealed a pronounced non-linear relationship (*P* for non-linearity = 0.005), indicating that these linear estimates do not fully capture the underlying exposure–response pattern. The formal non-linear test (based on the D1 statistic of multiple imputation) rejected the linear hypothesis (*P* = 0.005). Through two-stage Cox regression, the median of the inflection point determined in 20 multiple imputation datasets was 5.59 (95% CI: 4.78–6.06). When the CHG index was lower than 5.59, for every 1-unit increase, the stroke hazard increased by 117% (HR = 2.17, 95% CI: 1.37–3.44, *P* = 0.001); when the CHG index was ≥ 5.59, this association completely disappeared (HR = 1.00, 95% CI: 0.62–1.62, *P* = 0.996). The likelihood ratio test confirmed that the two-stage model was superior to the standard linear model (*P* = 0.005). This threshold effect could be visualized in both the restricted cubic spline curve and the segmented regression curve` (Figs. 2 and 3). The detailed results of the linear and two-piecewise Cox regression models are presented in Table 2.

**Table 2:** Sequential Cox regression models for the association between CHG index and new‑onset stroke

Outcome	Model 1 (age, sex) HR (95% CI)	Model 1 *P* value	Model 2 (+ lifestyle/socioeconomic) HR (95% CI)	Model 2 *P* value	Model 3 (fully adjusted) HR (95% CI)	Model 3 *P* value
Standard Cox regression						
CHG per 1-unit increase	1.84 (1.49–2.27)	< 0.001	1.84 (1.49–2.26)	< 0.001	1.48 (1.15–1.90)	0.003
Two-piecewise Cox regression						
Inflection point	–	–	–	–	5.59	–
CHG < inflection point	–	–	–	–	2.17 (1.38–3.42)	0.001
CHG ≥ inflection point	–	–	–	–	1.00 (0.62–1.62)	0.991
P for likelihood test	–	–	–	–	–	0.002

Association between the CHG index and new-onset stroke assessed by standard linear and two-piecewise Cox regression models under three sequential adjustment schemes. Model 1 was adjusted for age and sex; Model 2 additionally adjusted for marital status, education level, smoking, and drinking; Model 3 was fully adjusted for all covariates in Table 1. The two-piecewise model (threshold = 5.59) was fitted only within Model 3; dashes (–) indicate that the analysis was not applicable in the corresponding model. The likelihood ratio test compared the two-piecewise model with the standard linear model under Model 3. Results were pooled across 20 multiply imputed datasets

*Abbreviations*: *HR* Hazard ratio, *CI* Confidence interval

### Logistic regression analysis of new-onset stroke

As a secondary analysis, we further fitted a multivariable logistic regression model to assess the direction and magnitude of association. After full adjustment, each 1-unit increase in CHG was associated with significantly elevated odds of stroke (OR = 1.54, 95% CI: 1.17–2.03, *P* = 0.002). This estimate was directionally consistent with the Cox regression HR (1.48, 95% CI: 1.15–1.90, *P* = 0.003) and with the sharply elevated risk at lower CHG levels observed in the nonlinear piecewise models. Hence, the logistic analysis reinforces the robustness of the primary non-linear findings (Table 3).

**Table 3:** Multivariable logistic regression for factors associated with new-onset stroke

Variable	OR (95% CI)	*P* value
CHG index (per unit)	1.54 (1.17–2.03)	0.002
Age (per year)	1.01 (1.00–1.02)	0.064
Male (vs. Female)	0.97 (0.71–1.32)	0.827
Rural residence (vs. Urban)	0.96 (0.77–1.20)	0.717
Unmarried (vs. Married)	1.10 (0.83–1.46)	0.505
Education: Middle/High school (vs. Elementary)	1.22 (0.97–1.54)	0.082
Education: College or above (vs. Elementary)	1.23 (0.59–2.59)	0.583
Current smoker (vs. Non-smoker)	1.20 (0.90–1.60)	0.216
Current drinker (vs. Non-drinker)	1.12 (0.87–1.43)	0.372
Liver disease (vs. No)	1.47 (0.96–2.25)	0.074
Digestive disease (vs. No)	0.98 (0.78–1.22)	0.830
CES-D score (per unit)	1.04 (1.02–1.06)	< 0.001
Hypertension (vs. No)	2.10 (1.69–2.61)	< 0.001
Diabetes (vs. No)	1.17 (0.81–1.69)	0.393
Psychological treatment (vs. No)	2.10 (0.46–9.59)	0.338
BMI: Overweight (vs. Normal)	1.24 (0.95–1.62)	0.116
BMI: Obese (vs. Normal)	1.32 (0.90–1.94)	0.152

In the multivariable logistic regression model, the associations between CHG index, covariates, and new-onset stroke events were examined. Odds ratios (OR) and 95% confidence intervals (CI) are reported. The model was adjusted for age, gender, residence, marital status, education level, smoking, drinking, liver disease, digestive disease, CES-D score, hypertension, diabetes, psychological treatment, and BMI category. Results were pooled across 20 multiply imputed datasets

*Abbreviations*: *OR* Odds ratio, *CI* Confidence interval, *CHG* Cholesterol–high-density lipoprotein–glucose, *CES-D* Center for Epidemiologic Studies Depression Scale, *BMI* Body mass index

### Sensitivity analysis

We conducted a series of sensitivity analyses to verify the robustness of the results. All the results are summarized in Table 4. The detailed quartile cutoffs and baseline characteristics are presented in Supplementary Table S2. Firstly, the CHG index was divided into quartiles (Q1–Q4), with Q1 as the reference group. A fully adjusted Cox regression model was used to evaluate the relationship between each quartile and the occurrence of new-onset stroke. The results showed that the stroke risk of participants in the highest quartile (Q4) was significantly higher than that of Q1 (HR = 1.85, 95% CI: 1.30–2.62, *P* < 0.001); the risks of Q2 and Q3 also increased, but did not reach statistical significance (Q2: HR = 1.31, 95% CI: 0.89–1.93; Q3: HR = 1.30, 95% CI: 0.89–1.90). The trend test was marginally significant (trend *P* = 0.070), indicating an increasing trend in stroke risk as the CHG level increased. Secondly, the two-stage Cox regression further supported the existence of the threshold effect. With the inflection point of 5.59 determined by the minimum AIC, when CHG was less than 5.59, for every 1-unit increase, the stroke hazard increased by 117% (HR = 2.17, 95% CI: 1.37–3.44, *P* = 0.001); when CHG was ≥ 5.59, the association was no longer statistically significant (HR = 1.00, 95% CI: 0.62–1.62, *P* = 0.996), suggesting that the risk tended to saturate after the inflection point. Additionally, to rule out the possibility of reverse causation, we excluded participants who had experienced a stroke within the previous 2 years of follow-up. After repeating the Cox regression, the HR for continuous CHG was 1.44 (95% CI: 1.10–1.88, *P* = 0.009), the HR for Q4 vs. Q1 was 1.75 (95% CI: 1.21–2.54, *P* = 0.004), the HR on the left side of the inflection point was 2.20 (95% CI: 1.11–4.37, *P* = 0.026), and the results were highly consistent with the main analysis. To avoid a single reliance on the data-driven threshold, we also conducted a pre-defined median binary classification analysis (cut-off value 5.26). The results showed that the stroke risk in the high CHG group was significantly increased (HR = 1.34, 95% CI: 1.05–1.71, *P* = 0.022). Finally, as a secondary analysis, the multivariable Logistic regression also obtained a directionally consistent significant association (OR = 1.53, 95% CI: 1.16–2.03, *P* = 0.003), further confirming the robustness of the effect. All the results of the above sensitivity analyses were consistent with the main analysis, supporting the robustness of the positive association between the CHG index and the occurrence of new-onset stroke.

**Table 4:** Summary of sensitivity analyses for the association between CHG and new-onset stroke

	HR (95% CI)	*P* value
Primary Cox model		
Continuous CHG (per 1 unit)	1.48 (1.15–1.90)	0.003
Quartile analysis (Cox)		
Q2 vs. Q1	1.31 (0.89–1.93)	0.173
Q3 vs. Q1	1.30 (0.89–1.90)	0.184
Q4 vs. Q1	1.85 (1.30–2.62)	< 0.001
P for trend		0.070
Piecewise Cox (threshold 5.59)		
CHG < 5.59	2.17 (1.37–3.44)	0.001
CHG ≥ 5.59	1.00 (0.62–1.62)	0.996
Excluding first 2-year strokes		
Continuous CHG	1.44 (1.10–1.88)	0.009
Q4 vs. Q1	1.75 (1.21–2.54)	0.004
CHG < 5.59	2.20 (1.11–4.37)	0.026
CHG ≥ 5.59	1.17 (0.79–1.74)	0.432
Dichotomized at median (5.26)		
High vs. Low CHG	1.34 (1.05–1.71)	0.022
Logistic regression (secondary)		
Continuous CHG	1.53 (1.16–2.03)	0.003

All HRs are from multivariable Cox (or logistic) regression adjusted for age, sex, residence, marital status, education, smoking, drinking, liver disease, digestive disease, CES-D score, hypertension, diabetes, psychological treatment, and BMI. Results are pooled across 20 imputed datasets.

### Subgroup analysis

**Fig. 4:**
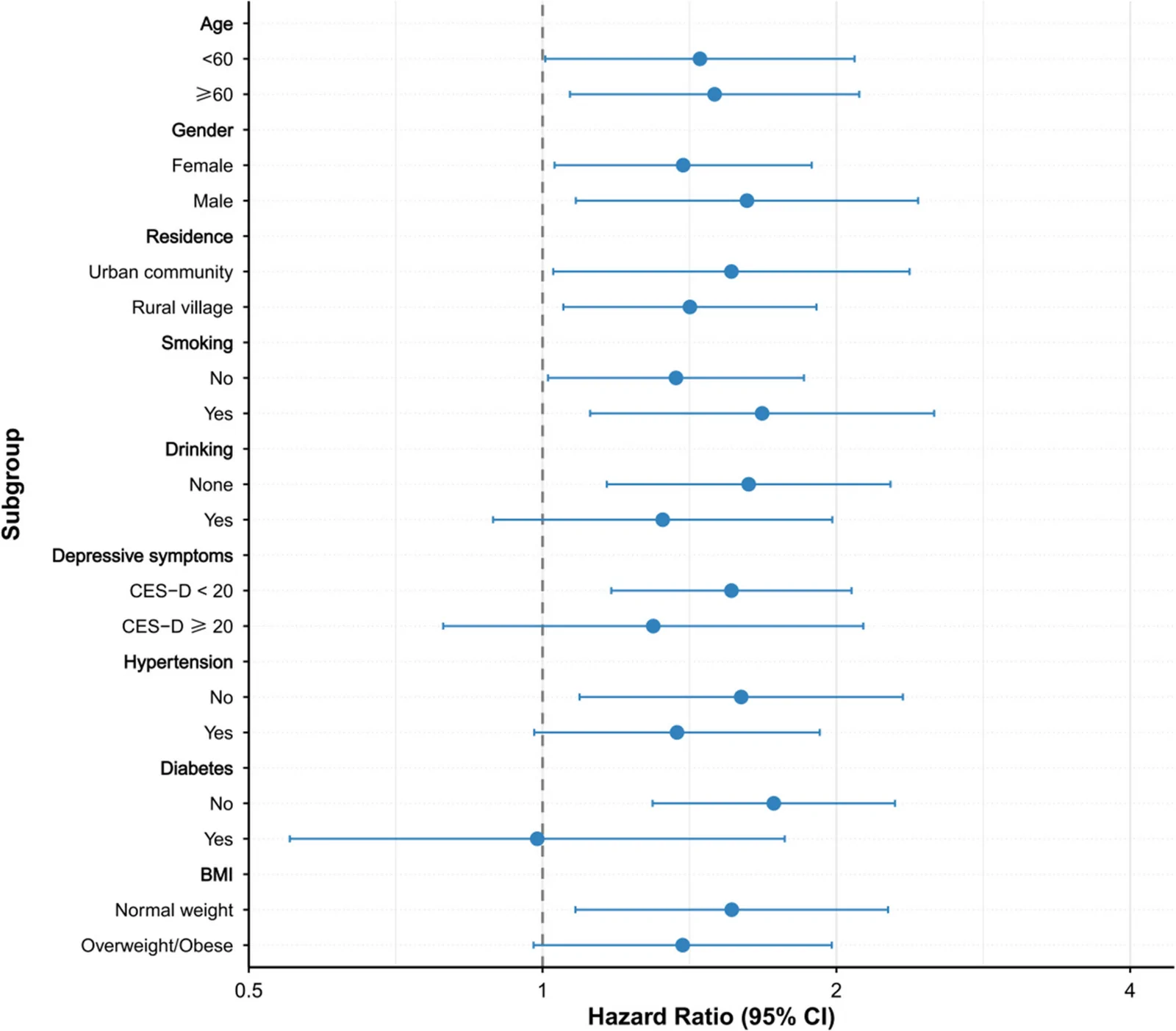
Forest plot of subgroup analysis of the association between CHG index and new‑onset stroke. All subgroup analyses were conducted using fully adjusted Cox regression models. Subgroups were stratified by age (< 60 vs. ≥60 years), sex (female vs. male), residence (urban community vs. rural village), education level (elementary school or below vs. middle school or above), smoking status (no vs. yes), drinking status (no vs. yes), history of hypertension (no vs. yes), history of diabetes (no vs. yes), BMI classification (normal weight vs. overweight/obese), and severity of depressive symptoms (CES-D < 20 vs. ≥20). Hazard ratios (HR) and 95% confidence intervals (CI) for the association between CHG index and new‑onset stroke in each subgroup are presented. All interaction terms between CHG index and subgroup variables were nonsignificant (all P for interaction > 0.05). Abbreviations: HR, hazard ratio; CI, confidence interval; CES-D, Center for Epidemiologic Studies Depression Scale

To evaluate the consistency of the association between the CHG index and the incidence of new-onset stroke across different populations, we conducted a subgroup analysis based on fully adjusted Cox regression, and stratified by age (< 60 years / ≥ 60 years), gender (female/male), place of residence (urban community/rural village), educational level (primary school and below/middle school and above), smoking status (no/yes), drinking status (no/yes), history of hypertension (no/yes), history of diabetes (no/yes), BMI classification (normal weight/overweight or obese), and severity of depressive symptoms (CES-D < 20 / CES-D ≥ 20). The results showed that the positive association between the CHG index and the incidence of new-onset stroke was generally consistent across all subgroups, and all HR point estimates were located to the right of the null line (HR = 1), without any directional reversal (Fig. 4).

Among those with age < 60 years, age ≥ 60 years, female, male, urban residents, rural residents, non-smokers, smokers, non-drinkers, no hypertension, hypertension, no diabetes, and normal weight, the association between CHG elevation and increased stroke risk was statistically significant (*P* < 0.05), with HR ranges ranging from 1.5 to 1.9. Among them, the association strength for males (HR close to 1.8) was slightly higher than that for females (HR approximately 1.5), and smokers were slightly higher than non-smokers. In drinkers and overweight/obese individuals, the point estimates still indicated an increased risk, but did not reach statistical significance, and their confidence intervals overlapped with the main results. The 95% confidence intervals of the diabetes group (Diabetes: Yes) and the severe depression symptom group (CES-D ≥ 20) crossed HR = 1, suggesting that the association between CHG and the incidence of new-onset stroke was not statistically significant in these subgroups. The confidence interval of the severe depression symptom group was wider, possibly related to the smaller sample size.

The interaction between CHG and each stratified variable was not statistically significant (all interactions *P* > 0.05), indicating that the effect of CHG did not show significant differences among different characteristic populations, and the predictive effect was consistently robust.

## Discussion

The sequential Cox regression analyses (Models 1–3) demonstrated that an increase in the CHG index was significantly associated with an elevated risk of stroke. This association remained robust after progressive adjustment for demographic, socioeconomic, lifestyle, and clinical confounders, with the HR decreasing from 1.84 to 1.48, suggesting that the association is not solely explained by the measured confounders. Logistic regression, as a secondary analysis, also yielded consistent results in the same direction, further validating the reliability of this association. This association exhibits a clear nonlinear threshold feature, and the nonlinear test reached statistical significance. After controlling for multiple confounding factors, this association remained stable. Additionally, in our analysis, we proposed a potential threshold pattern (CHG index = 5.59). When the index is below this turning point, for every one-unit increase in the CHG index, the risk of stroke increases significantly by 117% (HR = 2.17, *P* = 0.001); while when the index is above this turning point, the risk association disappears. This finding suggests that the CHG index has a robust association with new-onset stroke events in patients with depression, and its risk effect has clear segment-specific specificity. Moreover, when the CHG index was analyzed by quartiles, the highest quartile (Q4) was still associated with a significant increase in the risk of new-onset stroke, while the middle quartiles (Q2, Q3) did not show a significant increase, and the trend test was marginally significant (trend *P* = 0.070). This pattern is generally consistent with the nonlinear association observed in the preliminary analysis and is further verified through a sensitivity analysis after multiple imputation, supporting the reliability of our research results.

Previous studies have shown that depressive symptoms are closely associated with an increased risk of new-onset stroke [26, 27]. In the population with depressive symptoms, previous studies have found that metabolic indicators such as the atherogenic index of plasma (AIP), which are constructed based on lipid parameters, are significantly associated with the risk of new-onset stroke, suggesting that metabolic disorders are of great significance in depression-related strokes [28]. However, the metabolic abnormalities in people with depression are not limited to abnormal lipid metabolism; they are also widespread in terms of abnormal glucose metabolism [29]. The CHG index integrates total cholesterol, high-density lipoprotein cholesterol and fasting blood glucose, and can comprehensively reflect the lipid and glucose metabolism status. However, the CHG index has previously been mainly validated in populations with cardiovascular diseases or diabetes [14, 16], there is still a lack of evidence regarding its role in assessing the risk of new-onset stroke in the participants with depressive symptoms. Therefore, this study explored the relationship between the CHG index and new-onset stroke in the participants with depressive symptoms, and further evaluated its potential non-linear characteristics.

From the perspective of pathophysiology, as a chronic stress state, depression can over-activate the hypothalamic-pituitary-adrenal axis, induce chronic low-grade inflammation and autonomic nerve dysfunction [30, 31]. The excessive activation of the HPA axis leads to an increase in cortisol, which inhibits the insulin signaling pathway, induces or aggravates insulin resistance, and causes an increase in fasting blood sugar and total cholesterol levels. Insulin resistance can accelerate vascular endothelial dysfunction and also promote the formation of foam cells, promote the formation and instability of atherosclerotic plaques, and directly participate in the development of atherosclerosis [32, 33]. Meanwhile, the release of pro-inflammatory cytokines (such as IL-6, TNF-α) triggers chronic low-level inflammation, reduces the level of high-density lipoprotein cholesterol (HDL‑C), enhances the oxidative modification of low-density lipoprotein, and aggravates endothelial damage and oxidative stress [34]. In addition, autonomic nerve dysfunction, especially the continuous excitation of the sympathetic nerve, can further amplify the inflammatory response and metabolic abnormalities, and enhance the activation, aggregation and adhesion of platelets. At the same time, it is accompanied by inhibition of the fibrinolytic system and upregulation of coagulation factors (such as PAI-1), thereby increasing the risk of thrombosis [35]. The above abnormalities of lipid and glucose metabolism, inflammation, and imbalance of coagulation and fibrinolysis system can jointly lead to cerebrovascular endothelial dysfunction, enhance oxidative stress, and promote the formation and instability of atherosclerotic plaques, thus significantly increasing the risk of thrombosis, vascular embolism or rupture [36–38], all of these factors constitute the pathological basis of new-onset stroke. The CHG index, which integrates three key parameters of lipid and carbohydrate metabolism, shows a significant association with the risk of new-onset stroke in the participants with depressive symptoms, to some extent reflecting the multi-level interaction between depression and new-onset stroke.

Our study is the first to demonstrate a significant nonlinear threshold effect between the CHG index and the risk of new-onset stroke in participants with depressive symptoms. This result was consistently observed in both unadjusted analysis and the fully adjusted Cox model (Model 3), indicating a high degree of robustness of this effect in the presence of potential confounding factors. Specifically, the turning point determined by two-stage Cox regression was CHG index = 5.59. When the CHG index was below this critical value, for every one-unit increase, the risk of stroke would increase by 117% (HR = 2.17, *P* = 0.001); while when the CHG index was above this critical value, this association completely disappeared (HR = 1.00, *P* = 0.996), indicating that the risk tended to saturate after the turning point. The cumulative impact of metabolism may be partially reflected through this comprehensive metabolic indicator CHG. This research result emphasizes the importance of conducting clinical intervention when the CHG index is below 5.59, providing a theoretical basis for the clinical management of patients with depression. Additionally, the subgroup analysis results indicate that the positive association between the CHG index and the risk of new-onset stroke is generally consistent in each subgroup, and all HR point estimates are located to the right of the null line (HR = 1), without any directional reversal. It is notable that the significant association observed in individuals without diabetes, without hypertension, and with normal body mass index indicates that the CHG index may have particular significance as an early risk indicator before obvious metabolic complications occur. For individuals already suffering from these diseases, the predictive value of the CHG index may be partially weakened, which may be due to the dominance of these comorbidities in the risk of stroke. Although some point estimates did not reach statistical significance in certain subgroups (such as rural residents, drinkers, overweight/obese individuals, diabetes patients, and those with severe depressive symptoms (CES-D ≥ 20)), this may reflect the limited sample size or the dominant role of existing comorbidities. Moreover, the interaction effects in each subgroup were not significant (all interaction *P* > 0.05), indicating that the CHG index as a predictor showed consistency and universality in different subgroups of participants with depressive symptoms.

This study has several significant advantages. Firstly, based on the nationally representative large sample longitudinal data of CHARLS, this study effectively addressed the issue of missing covariates using multiple imputation methods, reducing selection bias and enhancing the credibility of the research results. Secondly, we used the Cox proportional hazards regression model as the main analysis, which made the effect estimation more accurate; at the same time, we supplemented it with Logistic regression as a sensitivity analysis to confirm the robustness of the conclusion. Moreover, by combining restricted cubic splines with two-stage Cox regression, this study identified the non-linear threshold relationship between the CHG index and new-onset stroke, and verified the stability of the turning point through the median of the multiple imputation dataset and Bootstrap resampling, thereby enhancing the clinical reference value of the study. Finally, we conducted a comprehensive subgroup analysis and sensitivity analysis (including excluding early stroke events, median binary classification and quartile analysis), further confirming the consistency of the CHG index in predicting stroke risk in the participants with depressive symptoms.

This study still has certain limitations. Firstly, the research subjects were limited to Chinese middle-aged and elderly people aged 45 and above. The applicability of the research conclusions in younger populations and other racial groups needs further verification. Secondly, the determination of stroke events mainly relied on self-reported questionnaires, which may have recall bias and misclassification bias; and due to data limitations, it was not possible to distinguish between ischemic and hemorrhagic strokes, and the risk factor spectra of these two types of strokes may be different. Thirdly, although multiple potential confounding factors have been adjusted, due to the limitations of the CHARLS database, information on some important variables, such as physical activity, glycosylated hemoglobin (HbA1c), C-reactive protein (CRP), LDL-C and triglyceride levels, was still lacking, thus making it impossible to completely rule out residual confounding. Fourthly, the CHG index was calculated based on a single baseline measurement and failed to reflect its dynamic effect over time; moreover, the time of stroke events was inferred based on self-reported follow-up waves and was not an exact clinical event date, which may lead to imprecision in time measurement.Fifthly, since the CHARLS dataset we used for multiple imputation does not contain detailed and unified variables for death time in the data files, we are unable to conduct a formal competing risk analysis such as the Fine-Gray subdistribution risk model. In the Cox model, participants who died during the follow-up period are treated as censored. Although the results from sensitivity analyses are robust, and the non-linear associations are consistent in quartile-based and segmented models, we cannot completely rule out the possibility that the competing risk from death slightly deviates from the reported hazard ratio. Future studies with complete specific etiology survival data should explicitly model death as a competing event in the models. Besides, because the CHG index was proposed after the CHARLS baseline data were collected, our results reflect a retrospective application of the formula and should be regarded as exploratory. Prospective studies are needed to validate the predictive utility of the CHG index. Finally, although the threshold effect was statistically significant, the risk attenuation above the inflection point may be partly due to the sparse data in the high CHG region, and its biological significance still needs to be further confirmed by larger sample and prospective studies. Future research can further verify and expand the findings of this study by including more precise stroke diagnosis information, supplementing more key confounding factors, using repeated measurement exposure indicators, and conducting multi-center long-term follow-up.

## Conclusion

This study found that in middle‑aged and older Chinese adults with depressive symptoms, an increase in the CHG index was significantly associated with an increased risk of new-onset stroke, and there was a significant nonlinear threshold relationship between the two. The turning point was located at a CHG index of 5.59. These findings suggest that the CHG index may serve as a potential risk indicator for new-onset stroke in middle‑aged and older Chinese adults with depressive symptoms, thereby helping to reduce the stroke burden of this group.

## Supplementary Information

Supplementary Material 1.

Supplementary Material 2.

## Data Availability

The data for this study were obtained from the CHARLS database (http://charls.pku.edu.cn/).
