# Supplementary material for: Association between the cholesterol-high-density lipoprotein-glucose index and new-onset stroke in middle-aged and older adults with depressive symptoms: a longitudinal cohort analysis
Source: Lipids Health Dis. 2026 Jul 18;25:213. doi: 10.1186/s12944-026-03001-5 (PMC13717998; doi:10.1186/s12944-026-03001-5)
Supplement: Supplementary file 1 [file 12944_2026_3001_MOESM1_ESM.docx]

Table S1. Baseline characteristics of excluded vs. included participants.
Values are presented as mean (SD) or median [IQR] for continuous variables and n (%) for categorical variables.
Missing data are shown as percentages. P values from t‑test / Kruskal‑Wallis / chi‑square test.

| **Variable** | **level** | **Total**  **(n=17705)** | **Excluded(n=12567)** | **Included(n=5138)** | **p** | **Missing** |
| --- | --- | --- | --- | --- | --- | --- |
| Age, Mean±SD |  | 58.50 (10.17) | 58.01 (10.43) | 59.67 (9.43) | <0.001 | 1.0 |
| gender (%) | Female | 9227 (52.1) | 6030 (48.0) | 3197 (62.2) | <0.001 | 0.0 |
|  | Male | 8478 (47.9) | 6537 (52.0) | 1941 (37.8) |  |  |
| residence (%) | Urban community | 7168 (40.5) | 5562 (44.3) | 1606 (31.3) | <0.001 | 0.0 |
|  | Rural village | 10537 (59.5) | 7005 (55.7) | 3532 (68.7) |  |  |
| Marital status, *n* (%) | Married | 15576 (88.0) | 11236 (89.4) | 4340 (84.5) | <0.001 | 0.2 |
|  | Separated,divorced,  widowed and never married | 2099 (11.9) | 1301 (10.4) | 798 (15.5) |  |  |
|  | Missing | 30 ( 0.2) | 30 ( 0.2) | 0 ( 0.0) |  |  |
| Education, *n* (%) | Elementary school or below | 8038 (45.4) | 5122 (40.8) | 2916 (56.8) | <0.001 | 0.1 |
|  | Middle school to high school | 8835 (49.9) | 6701 (53.3) | 2134 (41.5) |  |  |
|  | Vocational school, college and above | 818 ( 4.6) | 730 ( 5.8) | 88 ( 1.7) |  |  |
|  | Missing | 14 ( 0.1) | 14 ( 0.1) | 0 ( 0.0) |  |  |
| Smoking, *n* (%) | No | 10626 (60.0) | 7258 (57.8) | 3368 (65.6) | <0.001 | 0.8 |
|  | Yes | 6931 (39.1) | 5161 (41.1) | 1770 (34.4) |  |  |
|  | Missing | 148 ( 0.8) | 148 ( 1.2) | 0 ( 0.0) |  |  |
| Drinking, *n* (%) | No | 10716 (60.5) | 7359 (58.6) | 3357 (65.3) | <0.001 | 0.9 |
|  | Yes | 6823 (38.5) | 5045 (40.1) | 1778 (34.6) |  |  |
|  | Missing | 166 ( 0.9) | 163 ( 1.3) | 3 ( 0.1) |  |  |
| CHG (median [IQR]) |  | 5.28 [5.04, 5.57] | 5.29 [5.05, 5.58] | 5.27 [5.01, 5.53] | <0.001 | 41.0 |
| Liver disease, *n* (%) | No | 16831 (95.1) | 11969 (95.2) | 4862 (94.6) | <0.001 | 1.5 |
|  | Yes | 605 ( 3.4) | 373 ( 3.0) | 232 ( 4.5) |  |  |
|  | Missing | 269 ( 1.5) | 225 ( 1.8) | 44 ( 0.9) |  |  |
| Digestive disease, *n* (%) | No | 13735 (77.6) | 10185 (81.0) | 3550 (69.1) | <0.001 | 1.1 |
|  | Yes | 3780 (21.3) | 2206 (17.6) | 1574 (30.6) |  |  |
|  | Missing | 190 ( 1.1) | 176 ( 1.4) | 14 ( 0.3) |  |  |
| CES-D score (median [IQR]) |  | 7.00 [3.00, 12.00] | 5.00 [2.00, 7.00] | 14.00 [12.00, 18.00] | <0.001 | 9.4 |
| Hypertension, *n* (%) | No | 12933 (73.0) | 9237 (73.5) | 3696 (71.9) | <0.001 | 1.3 |
|  | Yes | 4535 (25.6) | 3126 (24.9) | 1409 (27.4) |  |  |
|  | Missing | 237 ( 1.3) | 204 ( 1.6) | 33 ( 0.6) |  |  |
| Diabetes, *n* (%) | No | 16344 (92.3) | 11593 (92.2) | 4751 (92.5) | <0.001 | 1.7 |
|  | Yes | 1062 ( 6.0) | 723 ( 5.8) | 339 ( 6.6) |  |  |
|  | Missing | 299 ( 1.7) | 251 ( 2.0) | 48 ( 0.9) |  |  |
| Psychological treatment, *n* (%) | No | 17443 (98.5) | 12341 (98.2) | 5102 (99.3) | <0.001 | 1.2 |
|  | Yes | 42 ( 0.2) | 27 ( 0.2) | 15 ( 0.3) |  |  |
|  | Missing | 220 ( 1.2) | 199 ( 1.6) | 21 ( 0.4) |  |  |
| BMI (median [IQR]) |  | 23.10 [20.79, 25.74] | 23.32 [21.03, 25.92] | 22.62 [20.31, 25.33] | <0.001 | 23.0 |

Included: participants meeting age ≥45, stroke‑free at baseline, CES‑D ≥10, and with available follow‑up stroke data. Excluded: all other participants (including those with missing CES‑D or screening variables).
