# Supplementary material for: Association between the cholesterol-high-density lipoprotein-glucose index and new-onset stroke in middle-aged and older adults with depressive symptoms: a longitudinal cohort analysis
Source: Lipids Health Dis. 2026 Jul 18;25:213. doi: 10.1186/s12944-026-03001-5 (PMC13717998; doi:10.1186/s12944-026-03001-5)
Supplement: Supplementary file 2 [file 12944_2026_3001_MOESM2_ESM.docx]

Table S2. Baseline characteristics and stroke events by CHG quartiles.
Quartile cut‑offs are based on the pooled CHG distribution from the first imputed dataset.
Data are presented as mean ± SD or n (%).

| **Quartile** | **CHG range** | **N** | **Events, n (%)** | **Age, mean ± SD** | **CHG, mean ± SD** | **Male, %** |
| --- | --- | --- | --- | --- | --- | --- |
| Q1 | ≤ 5.00 | 1,266 | 73 (5.8%) | 60.2 ± 9.7 | 4.82 ± 0.17 | 41.2 |
| Q2 | (5.01, 5.26] | 1,283 | 90 (7%) | 59.2 ± 9.5 | 5.13 ± 0.07 | 38.8 |
| Q3 | (5.26, 5.52] | 1,256 | 95 (7.6%) | 59.4 ± 9.4 | 5.38 ± 0.07 | 35.5 |
| Q4 | > 5.52 | 1,333 | 163 (12.2%) | 59.9 ± 9.2 | 5.86 ± 0.36 | 35.6 |
| CHG quartile cut‑offs (based on the first imputed dataset): Q1 ≤ 5.00, Q2 5.01–5.26, Q3 5.27–5.52, Q4 > 5.52. Data are presented as mean ± SD or n (%). | | | | | | |
